# A Prospective Study of the Incidence and Correlated Factors of Post-Stroke Depression in China

**DOI:** 10.1371/journal.pone.0078981

**Published:** 2013-11-18

**Authors:** Wei-Na Zhang, Yong-Hui Pan, Xiao-Yu Wang, Yue Zhao

**Affiliations:** Department of Neurology, the First Clinical Medical College of Harbin Medical University, Harbin, PR China; University of Nebraska Medical Center, United States of America

## Abstract

**Background:**

Post-stroke depression (PSD) is commonly observed among stroke survivors. However, statistical analysis of such data is scarce in developing countries. The purpose of this study is to examine the incidence of PSD and its relationship with stroke characteristics in China.

**Methods:**

This was a prospective hospital-based study. Stroke patients were assessed within two weeks after acute ischemic stroke onset and then reevaluated at three months. Hamilton Depression Scale (HAMD) was used for screening depression (PSD). Subjects with HAMD score of ≥7 were further assessed with the World Health Organization Composite International Diagnostic Interview. Stroke severity was measured by the National Institutes of Health Stroke Scale (NIHSS). Stroke outcome was measured by the modified Rankin Scale (mRS).

**Results:**

One hundred and two stroke patients were recruited, only ninety-one patients completed del period (men = 53, 63.74%), with mean age 60.0±10.4 years (range, 34–82 years). The incidence of PSD was 27.47% two weeks after stroke. The occurrence of PSD was unrelated with age, stroke type, stroke lesion and the history of disease. In univariate analysis gender, PSD was correlated with female gender. In multivariate logistic regression analysis, poor stroke outcome (mRS≥3) (OR 12.113, CI 1.169 to 125.59, *P*<0.05) was the important predictors of PSD.

**Conclusions:**

The study indicated that gender, functional dependence and stroke outcome are determinants of PSD occurrence during the first 2 weeks after stroke in China.

## Introduction

Post stroke depression (PSD) is a common complication of stroke that negatively interferes with outcome of stroke patients. Patients with PSD has more functional disability [1], poorer rehabilitation outcomes [2], reduced quality of life and increased mortality [3]. According to previous published data [4], mainly from developed countries, PSD has a high prevalence among stroke patients, ranging from 20 to 50%. The report also indicate that depression persists 3–6 months after stroke. In China, depression is one of the major mental disorders. In general population, it has a prevalence of about 2.0% [5]. However, regarding to the prevalence of PSD among stroke patients in China, there are no data available. The purpose of this study is to fill in such blank spot, and further analyze risk factors that correlate with PSD occurrence.

## Subjects and Methods

### 1. Subjects

This research was conducted in the First Affiliated Hospital of Harbin Medical University. Stroke patients were recruited from April 2010 to July 2011. The diagnosis of stroke was supported in every patient by computed tomography scanning and/or magnetic resonance imaging. Patients with a history of hearing disturbance, visual impairment, aphasia, comprehension disorders and refusal to consent were excluded. The protocol of this study was approved by the Medical Ethics Committee of the First Affiliated Hospital of Harbin Medical University, and written informed consent was obtained from all patients.

### 2. Clinical characteristics

Following information was collected for each patient: baseline demographics (age, gender, marital status, educational status, occupation, and economic status), stroke severity measured by the National Institutes of Health Stroke Scale [NIHSS] [6], stroke type according to Oxfordshire Community Stroke Project Classification [7], and stroke outcome measured by the modified Rankin scale [mRS] at two weeks and three months after stroke [8].

### 3. Depression screening tools

The subjects were screened for depressive symptoms using the 17-item Hamilton depression Rating Scale (HAMD) questionnaire at two weeks and three months. Subjects with a HAMD depression score of 7 or more were requested to be referred to trained researchers for further evaluation. Subjects with HAMD score of ≥7 were further assessed with version 3.0 of the World Health Organization Composite International Diagnostic Interview, as the diagnostic criteria of depression after stroke.

#### 3.1 Hamilton depression Rating Scale

HAMD is a validated method for measuring depression degree [9]. The questionnaire was administered according to the Interview Guide for the Hamilton Rating Scale, the purpose of which is to standardize the questions. The questionnaire has a scale that classifies degrees of depression according to these criteria: score lower than 6 – without mood swings/normal; from 7 to 17 – slightly depressed; from 18 to 24 – moderately depressed; above 25 – seriously depressed.

#### 3.2 World Health Organization Composite International Diagnostic Interview

The World Health Organization (WHO) Composite International Diagnostic Interview (WHO-CIDI 3.0) includes a screening module and 40 sections that focus on diagnoses (22 sections), functioning (four sections), treatment (two sections), risk factors (four sections), socio-demographic correlates (seven sections), and methodological factors (two sections). Innovations compared to earlier versions of the CIDI include expansion of the diagnostic sections, a focus on 12-month as well as lifetime disorders in the same interview, detailed assessment of clinical severity, and inclusion of information on treatment, risk factors, and consequences [10].

### 4. Statistical analysis

**Figure 1:**
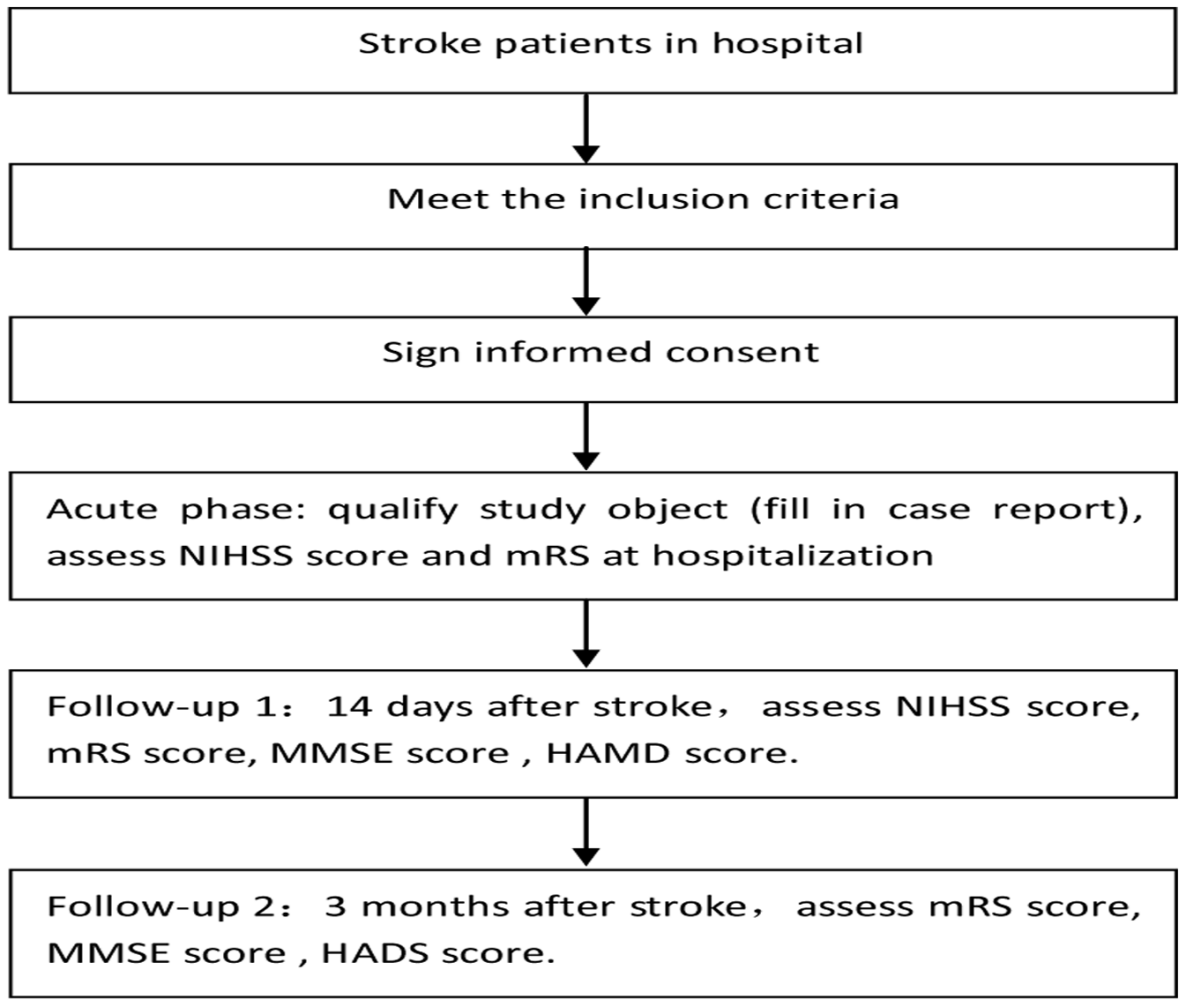
Research Process.

Wilcoxon rank sum test was used to analysis monofactorial measurement data. The monofactorial enumeration data were compared with χ^2^ test, and then the results were analyzed with multivariate analysis by non-conditional Logistic regression analysis. *P*<0.05 was considered as statistical significant. All the statistical analyses were performed with SAS9.2(Figure 1).

## Results

### 1. The incidence of PSD in 91 patients

In this study, 91 stroke patients were recruited. At two weeks after stroke, 25 cases showed depression. and incidence of PSD was 27.47%. At three months, 6 cases showed depression, and the incidence at this timepoint was 6.59%. The depression constituent ratio after two weeks were: light depression 22 cases, the proportion were 88%; medium depression 3 cases, the proportion was 12%, the severe depression 0 case.

### 2. The correlated factor of PSD

We include following factors in the study: gender, age, culture degree, occupation, income, the risk factor of cerebrovascular disease, stroke type, stroke localization, level of neurological impairment, self-care ability, et al (Table 1, 2). We found that gender was the only one factor which had statistical significance across mono-factor analysis (female is a risk factor, OR value is 3.1483, P<0.05). NIHSS score, mRS score, and MMSE score compared using Mann-Whitney U rank sum test. There were no statistically significant differences (Table 3). By stepwise logistic regression analysis of non-conditions, we concluded that the significant factors were gender and mRS score (Table 4). According to linear correlation analysis, we concluded that the correlation coefficient between mRs score and HAMD score was 0.99707, it prompt the two scores offered strong positive correlation, HAMD score was gradually cut down along with the decrease of mRS score; For those whose mRS score was higher, it is HAMD score was high (Table 5). Other demographic and stroke variables did not influence level of depression.

**Table 1:** Comparison of gender, age, culture degree, income between the two groups.

Factor		Control group (n = 66)	Depression group (n = 25)	OR95%CI	χ^2^ value	P value
Gender	male	47	11	0.3176 (0.1225–0.8235)	5.7453	0.0165
	female	19	14	3.1483 (1.2144–8.1622)	5.7453	0.0165
Age	≤44	6	0	0.1825 (0.0099–3.3617)	2.4064	0.1208
	44–60	32	11	0.8348 (0.3309–2.1065)	0.1447	0.7036
	≥60	28	14	1.7273 (0.6826–4.3707)	1.3299	0.2488
Culture degree	high	12	3	0.6136 (0.1577–2.3880)	0.4978	0.4805
	middle	39	12	0.6391 (0.2533–1.6123)	0.8954	0.3440
	low	15	10	2.2667 (0.8458–6.0741)	2.6851	0.1013
Occupation	Physical labor	16	4	0.6250 (0.1860–2.1005)	0.5779	0.4471
type	mental labor	16	4	0.6250 (0.1860–2.1005)	0.5779	0.4471
	Retirement and no-occupation	34	17	2.0000 (0.7588–5.2712)	1.9782	0.1596
Income	inferior	31	13	1.2231(0.4867–3.0740)	0.1817	0.6699
	middle	32	11	0.8348(0.3309–2.1065)	0.1447	0.7036
	high	3	1	0.8750(0.0867–8.8283)	0.0127	0.9103

**Table 2:** Comparison of risk factors of cerebrovascular disease, mental disorders, stroke type and localization.

Type		Depression group	Control group	OR95%CI	χ^2^	P
Diabetes	no	56	24			
	yes	10	1	0.2333(0.0283–1.9256)	2.0985	0.1474
Hypertension	no	24	7			
	yes	42	18	1.4694(0.5369–4.0216)	0.5585	0.4549
Hyperlipemia	no	46	20			
	yes	20	5	0.5750(0.1892–1.7478)	0.9554	0.3284
Cerebrovascular disease	no	44	16	2.8110(0.1401–56.3821)	1.1622	0.2810
	yes	22	9	0.3557(0.0177–7.1353)	1.1622	0.2810
Mental disorders	no	25	63	2.4000(0.2742–21.0048)	0.6545	0.4185
	yes	0	3	0.4167(0.0476–3.6467)	0.6545	0.4185
Stroke type	no	24	60	2.8110(0.1401–56.3821)	1.1622	0.2810
	yes	1	6	0.3557(0.0177–7.1353)	1.1622	0.2810
Stroke localization	left	26	9	1.1556(0.4450–3.0008)	0.0873	0.7677
	right	26	9	1.1556(0.4450–3.0008)	0.0873	0.7677
	bilateral	2	3	0.2292(0.0359–1.4627)	2.7785	0.0955
	middle	12	4	1.1667(0.3380–4.0266)	0.0589	0.8082

**Table 3:** Comparison of the score for the extent of neurological impairment, score of mRs and MMSE.

	Depression group	Control group	Wilcox on W	P
NIHSS score	53.00	43.35	1325.0	0.0713
mRS score	45.50	46.18	1137.5	0.9147
MMSE score	48.96	44.88	1224.0	0.5078

**Table 4:** The results of multiple stepwise logistic regression analysis.

Factor	β	S_b_	Waldχ^2^	P	OR	OR95%CI
Gender	1.1357	0.5172	4.8220	0.0281	3.113	1.130–8.579
mRS score						
4-0	2.0643	1.3055	2.5003	0.0138	7.880	1.610–101.799
3-0	2.4943	1.1930	4.3713	0.0365	12.113	1.169–125.529
2-0	0.2576	1.2333	0.0436	0.8345	1.294	0.115–14.511
1-0	−0.021	0.6163	0.0011	0.9731	0.979	0.293–3.278

**Table 5:** Correlation analysis between HAMD score and mRS score.

	HAMD score	mRS score
HAMD score	0.00100	0.007070.0029
mRS score	0.007070.0029	0.00100

## Discussion

Post-stroke depression is a frequent complication after stroke. It can interfere the function recovery of stroke patients and effect their quality of life. As Chinese Depressive Disorder Prevention and Treatment Guidelines in 2007 pointed out, PSD is the kind of disease with high incidence, high recurrence rate,and high mutilation rate.

In this study, PSD prevalence rate was 27.47% in the early post-stroke period. This is much lower than the prevalence of PSD in developed country, which was reported 40–50% [11], but more in line with some Asian countries' findings [12], [13]. At present, despite the abundant available literature, it is still difficult to define the true prevalence rate of PSD. This variability between studies arises not only from the methodological problems of the investigations but also from the complexity in recognizing, assessing, and diagnosing depression [14]. A study in Norway summarized that the prevalence of depressive disorder and depressive symptoms in the acute phase ranged widely from 5% to 54% [15]. Some studies found the prevalence of PSD was 44.6% in Portugal, 28% at the acute phase of stroke and 56% 4 months later in UK [16], [17]. Although there was considerable variation in the reported frequency of depression after stroke across individual studies. A meta-analysis has estimated the pooled frequency of PSD at 33%, even with relevant differences across studies [18]. In particular, the pooled estimate from the population-based studies was equal in the acute and medium-term phases(33%), with a slight increase to 34% in the long –term phase of recovery after stroke. Moreover, there were only slight differences in the pooled frequencies in the hospital-based (acute 36%, medium-term 32%, and long-term 34%) and rehabiliation-based studies (acute 30%, medium-term 32%, and long-term 34%) over time. In China, high levels of social network and psychological support for the elderly within Asian families may help reduce the incidence of depression after stroke. There are also less life events and psychosocial stressors in elderly Asian population. Another crucial point is that unlike in developed countries, most of our survivors went back home rather than to nursing homes or other institutions. Regarding to the severity of PSD, most patients were suffered from mild depression (88%). The severity rate of depression is lower than previous literatures too. Apart from prevalence of minor strokes, the long duration of stroke (mean duration of follow-up was 18 months) would have enabled survivors to adopt certain coping strategies to deal with depression. As well as this may be due to the selection of patients, as a large proportion of patients in this study have mild stroke, which may lead to less incidence of PSD and less severe depression.

There was no significant relationship between age and PSD,which was not in accordance with previous studies. Some reports showed that patients were prone to be troubled with PSD in age group 40–50, the incidence decreased over 60 years [19]. However, Naess found that stroke patients in age group 15–49 had lower PSD incidence than elderly patients in 196 stroke cases [20]. But some data suggest that there was no significant differences between various age groups [21]. The conflict conclusion may be drawn because different group of patients were chosen to be studied in different centers. Therefore, in future research, we look forward to a large sample, multi-center study, and should do comparison according to age hierarchies. In this study we found that, compared to male, the incidence of occurring PSD was higher in female. This is consistent with Paolucci's and Cassidy's finding [22], [23]. It may be because female patients were more vulnerable to psychological and social stress factors, and then result in physical, psychological balance disorder. But Kulkantrakorn thought that male PSD had unfavorable prognosis compared to female [13]. This may be explained by the nature of depression in this subgroup. As in other Asian developing communities, Thai males are predominant as a leader in family. If they are disabled due to stroke, the psychological impact on them is probably much more than that of female. As well as this result may be associated with the source of cases, the difference of male-female constituent ratio and so on, it was subject to large sample, multi-center statistics to do further study. About the relationship of PSD and cognitive function, there was not significant relationship between cognitive function and the occurrence of PSD in our study. But most of study suggested the patients who had cognitive functional impairment after stroke were more prone to get PSD, Naiushima thought that the MMSE score of PSD patient was generally lower than those who weren't depressed [24]. And in china some researchers came to the similar conclusion as well [25]. This study did confirm the correlation of stroke severity and PSD. In multiple stepwise logistic regression analysis, we found mRS was a risk factor of PSD, but the confidence interval was too wide, the conclusion should be confirmed by large sample data. This conclusion had also been confirmed in many studies. In our study, we also did a correlation analysis about mRS and HAMD score, there showed a strong positive correlation between the two facets. With the decline of mRS, HAMD score was also decreased. Severe physical disability after stroke had been found to be consistently associated with increased PSD risk [20], [22]. They had confirmed that moderate or severe disability may increase the risk of PSD by 20%. As well, severe disability may be linked to large lesions involving mood processing regions in the brain, Patients with severe disability may develop depression owing to great concern over social consequences [26].This conclusion needed further study to confirm, which should have large sample and multi-observation points.

In our study, there won't show that PSD was related to educational level, occupation type, household income, personality, past history and so on. Some researchers suggested the higher educational level was, the lower incidence of PSD was [27]. About the relationship between personality and PSD, Storor found patients with neurosis and psychological disorders were more prone to get depression [28].

With regard to the relation between PSD and stroke position, there was no conspicuous relationship showed in our study. Singh suggested that the lesion in left hemisphere close to frontal pole had specific correlation with PSD degree [9]. The same conclusion was found in study at home [29]. This conclusion needs further study to confirm.

Our study had several limitations. Its small sample size gives rise to high imprecision in some estimates. This would be minimized with a larger sample size. In addition, we excluded patients with severe aphasia because they could not complete the evaluation, and this might limit the generalization of our findings.

In conclusion, our study showed that the prevalence of PSD reached its peak value two weeks after stroke. Depression could be associated with burden caused by change of social and family role. One othe other hand, depression can conversely impact on activeness of rehabilitation and functional dependence. To stop such vicious circle, it requires early detecting of patients with high risk of developing PSD. Our study showed that female patients with severe stroke have the highest risk of suffering PSD. Therefore, more attentions and support should be provided to this group of patient.
